# 24-Month-Olds’ Selective Learning Is Not an All-or-None Phenomenon

**DOI:** 10.1371/journal.pone.0131215

**Published:** 2015-06-22

**Authors:** Annette M. E. Henderson, Susan A. Graham, Vanessa Schell

**Affiliations:** 1 School of Psychology, The University of Auckland, Auckland, New Zealand; 2 Department of Psychology, University of Calgary, Calgary, Alberta, Canada; Beijing Normal University, CHINA

## Abstract

Evidence that children maintain some memories of labels that are unlikely to be shared by the broader linguistic community suggests that children’s selective learning is not an all-or-none phenomenon. Across three experiments, we examine the contexts in which 24-month-olds show selective learning and whether they adjust their selective learning if provided with cues of in-context relevance. In each experiment, toddlers were first familiarized with a source who acted on familiar objects in either typical or atypical ways (e.g., used a car to mimic driving or hop like a rabbit) or labeled familiar objects incorrectly (e.g., called a spoon a “brush”). The source then labeled unfamiliar objects using either a novel word (e.g., *fep*; Experiment 1) or sound (e.g., *ring*; Experiments 2 and 3). Results indicated that toddlers learnt words from the typical source but not from the atypical or inaccurate source. In contrast, toddlers extended sound labels only when a source who had previously acted atypically provided the sound labels. Thus, toddlers, like preschoolers, avoid forming semantic representations of new object labels that are unlikely to be relevant in the broader community, but will form event-based memories of such labels if they have reason to suspect such labels will have in-context relevance.

## Introduction

Preschoolers are selective word learners, failing to learn new word-referent links that are unlikely to be shared by the broader linguistic community [1–4]. Although preschoolers do not form semantic representations of unconventional word-referent links, recent evidence suggests that preschoolers do remember some aspects of the labeling event as long as they have some reason to suspect the link might be relevant within the present context [5]. Thus, preschoolers’ selective learning is not an all-or-none phenomenon. In the present studies we address the issue of selective learning in 24-month-olds with specific focus on the contexts in which toddlers do and do not demonstrate selective learning. Specifically, we ask whether toddlers avoid forming semantic representations of unconventional labels, but demonstrate some memory for such labels if given evidence of in-context relevance.

An impressive body of empirical research has demonstrated that children are highly selective word learners [1–4]. Preschoolers avoid learning new object labels from sources who appear to be ignorant or uncertain of an object’s name [6], who have been shown to provide inaccurate object labels [7–11], who offer opinions that differ from the majority [12], who are novices [13,14], or who are unfamiliar [15]. Preschoolers’ selective learning is so pervasive that it has been demonstrated across a variety of contexts and domains of knowledge [2,16–18]. For example, Koenig and Harris [19] found that preschoolers would not learn the function of a novel object from a source who had previously provided incorrect object labels (see also [8]). Furthermore, studies demonstrate that selective learning emerges early in development [20–24]. For example, Koenig and Woodward [21] demonstrated that 24-month-olds would not learn a new word-referent link from a source with a history of providing inaccurate object labels (but see [22]). Similarly, Brooker and Poulin-Dubois [20] found that 18-month-olds were less likely to learn words from, or imitate the actions of, a source that had previously mislabeled familiar objects. Further, Zmyj et al. [23] revealed that 14-month-old infants were less likely to imitate the actions of a source that had previously acted on familiar objects in atypical ways. Thus, the evidence suggests that children as young as 14 months of age are selective consumers of information.

The mounting evidence surrounding children’s selective word learning clearly demonstrates that information in the word learning context, and more specifically information about the source who is providing a new word-referent link, influences whether children will learn a new word. Selective learning is a reasonable strategy in contexts like those reviewed above because language is a conventional communicative system; words are only successful communicative tools when their meanings are known and used by the members of a particular linguistic community [25,26]. Thus, the rationale for this selective word learning seems to be to avoid learning word-referent links that are unlikely to be shared by the broader linguistic community [1–4].

Recent evidence from a handful of studies raises the intriguing possibility that children’s selective word learning is not an all-or-none phenomenon—children will show some learning of new object labels that are unlikely to be broadly shared if there is reason to suspect the label might have in-context relevance [5,21,22,27]. In one such study, Sabbagh and Shafman [5] demonstrated that 4-year-old children showed some evidence of learning a new word-referent link provided by an ignorant speaker when they were asked an “episodic” comprehension test question (i.e., “Which one did I say is the modi?”), but not when they were asked a “semantic” comprehension test question (i.e., “Which one of these things is the modi?”), the type of question typically used in studies assessing children’s selective word learning. This finding suggests that, when preschoolers are presented with a new word-referent link that is unlikely to be shared by the broader linguistic community, they do not form semantic representations of such links, but do form a source-specific association between the labeling episode and the person providing the word-referent link. Critically, children’s source-specific associations in this study were short-lived; children did not remember the word-referent link when they were asked the episodic question after a brief delay. In a similar vein, Koenig and Woodward [21] demonstrated that 24-month-olds showed some memory for new word-referent links provided by an inaccurate source, but that their memory of such links quickly dissipated and would not transfer to a second speaker. These findings provide evidence that children do not completely ignore information provided by ignorant and inaccurate speakers and thus, raise questions concerning the processes underlying children’s selective learning.

The body of evidence surrounding children’s selective learning is consistent with the possibility that children might have different “modes of learning” depending on whether, or not, a new piece of information is likely to be relevant within the broader community. In the case of new word-referent links that are likely to be shared by the linguistic group and thus, likely to be relevant to future conversations, children engage their word learning resources and form a semantic representation of the word-referent association. However, if children have reason to suspect that a new word-referent link is unlikely to be shared by the linguistic group, as would be the case with links provided by ignorant or unreliable speakers, they enter a different learning mode in which they may form an event-based memory trace of the labeling episode, but not a semantic representation. In line with this possibility, Sabbagh and Shafman [5] propose that children use a “semantic-specific gating strategy” when they encounter word-referent links that are unlikely to be shared by the broader linguistic community. Such a strategy would allow children to encode potentially important aspects of the labeling event, such as who provided the irrelevant word-referent link, without forming a semantic representation of the link. In so doing, children equip themselves with a tool that might be relevant within the present context, perhaps a “local” understanding with the ignorant speaker about how to refer to a particular object, without the risk of acquiring a word-referent link that is unlikely to have relevance in the broader linguistic community (see also [5]).

While the intriguing possibility of two modes of learning fits with the selective learning literature (i.e., [5,21]), the range of contexts in which young children recruit semantic versus event-based learning remains unexamined. The present research was conducted to address this gap in the literature in a number of ways. Firstly, Experiment 1 was conducted to extend our understanding of the range of contexts in which toddlers would demonstrate selective learning. Previous studies have shown that children younger than 3 years of age demonstrate selective learning [20–23], however these studies have focused on toddlers’ selective learning from sources that were either inaccurate or ignorant. Thus, in Experiment 1, we examined whether toddlers would form semantic representations of (i.e. learn) new object labels provided by a source who had previously acted on objects in incorrect ways. To address this question, we asked whether 24-month-olds would be less likely to learn new object labels that had been provided by a source who had been shown either to use or label objects incorrectly (e.g., a source who pretended to brush her hair with a spoon or labeled a spoon a “brush”), than new labels provided by a source who had been shown to use and label objects correctly (e.g., labeled a spoon “a spoon” and pretended to eat soup with it). We expected that familiarizing toddlers to a source who uses objects incorrectly or a source who provides inaccurate object labels would provide toddlers with reason to doubt the conventionality of the labels provided by the source and as a result, toddlers would be less likely to demonstrate word learning in these cases than would toddlers who were familiarized to a source who had a history of being accurate and using objects in the appropriate ways.

In Experiment 2 we examine whether 24-month-olds’ selective learning is an all-or-none phenomenon. To address this question, we drew upon research investigating the types of symbols that infants will accept as names for objects. Initially, infants are symbolically open—accepting a wide array of symbols as object labels. However, this symbolic openness begins to narrow toward the end of the second year of life when toddlers begin to accept only words as object names [28–30]. Toddlers’ symbolic restrictiveness suggests that they do not form semantic representations of sound-object associations. However, it remains unclear whether toddlers would form event-based memories of the labeling event, particularly if they were given some reason to think that the association might be relevant to the current context. Thus, in Experiment 2 we ask whether toddlers would demonstrate some learning of a sound-object association if they were given reason to suspect that the association might be relevant within the present context. Experiment 3 was a follow-up experiment designed to directly test the extent to which toddlers’ willingness to accept sound-object mappings is driven by contextual relevance. Taken together, the three experiments reported here extend the literature by examining the conditions under which toddlers demonstrate selective word learning and offer the first investigation of whether toddlers, like preschoolers, show evidence of learning new object labels that are unlikely to be shared by the broader linguistic community in contexts in which such labels might have relevance.

## Experiment 1

### Method

#### Participants

Seventy-two 24-month-olds were included in the final sample. Participants were from homes in which English was the primary spoken language, were from varied socioeconomic backgrounds, and were primarily Caucasian (although the latter two factors were not formally assessed). Equal numbers of males and females were randomly assigned to one of three groups: **Typical Source** (*n* = 24; *M* = 24.11 months; *SD* = 1.01), **Atypical Source** (*n* = 24; *M* = 24.01 months; *SD* = .81), and **Inaccurate Source** (*n* = 24; *M* = 23.88 months; *SD* = 1.02). Toddlers did not differ in age or productive vocabulary size (as assessed by the MCDI: Words and Sentences, [31]) across the three groups (*p-*values > .51). Twenty-two additional toddlers were tested but were excluded from the final sample due to failure to complete the study or fussiness (*n* = 17) and experimenter error (*n* = 5).

#### Stimuli and Materials

Three familiar objects were used for the Familiarization Phase: a spoon, a cup, and a car. Four novel objects (i.e., a soft dog toy, a rubber dog toy, a loofah, and an atypical ball) were used during the novel label training and test trials. The novel objects were separated into two sets (one set per round). For each novel object in each set, there was a second object of like kind that differed only in colour (used for the generalization trial). A camera was mounted on the wall facing the infant.

#### Procedure

This research was approved by the Conjoint Faculties Research Ethics Board (CFREB) at the University of Calgary. We have followed CPA guidelines for the ethical treatment of participants. Participants first participated in a brief warm-up period in which the toddler was familiarized to the experimenter who would be running the session and the parent (or guardian) provided written and verbal informed consent on behalf of their child. After the warm-up period, participants were escorted into the room in which the experimental session was completed. Participants were seated at a table, with their parent seated beside them and the experimenter seated across from them. Toddlers in all groups participated in the following three phases: **familiarization, label training**, and **label test**.

#### Familiarization Phase

This phase was used to establish whether the experimenter behaved in a way that was typical, atypical, or inaccurate, depending on the group to which toddlers were assigned. The experimenter labeled each of the three familiar objects (e.g., a spoon) one at a time and provided either a typical or atypical function for the object (e.g. “I eat soup with this spoon” or “I brush my hair with this spoon”) or an inaccurate label (e.g., “This is a brush”, in reference to a spoon). Functions or inaccurate labels were repeated three times for each of the objects. See Table 1 for a list of the objects, actions, and inaccurate labels. Following this phase, the experimenter proceeded directly to the Label Training Phase.

**Table 1:** Typical actions, atypical actions, and inaccurate labels for each familiar object.

	Typical Actions	Atypical Actions	Inaccurate Labels
Spoon	“I use this spoon to eat soup”	“I use this spoon to brush my hair”	“This is a brush”
Cup	“I use this cup to drink”	“I use this cup to clean”	“This is a phone”
Car	“I make the car drive fast”	“I make the car hop like a rabbit”	“This is a ball”

#### Label Training Phase

The experimenter began this phase by introducing toddlers to one pair of novel objects. For each pair, one object was designated the target object and one the distractor object. The object designated the target and the order in which the target and distractor objects were presented were counterbalanced across toddlers.

When presenting the target object, the experimenter labeled the object with a novel count noun (“Look at what I have! *Fep*. That’s what we call this one!”). This type of labeling frame is similar to frames that have been used in past work (e.g., [29,32]) and was chosen to enable comparisons across all three of the experiments reported in this manuscript. The labeling phrase was repeated three times for each toddler. When presenting the distractor object, toddlers’ attention was drawn to the object in a similar manner (i.e., “Look at what this is! See what I have!”), but no label was provided. Following the introduction of each object, the experimenter passed the object to the infant for 15 seconds of exploration.

#### Label Test Phase

The test phase consisted of an extension trial and a generalization trial. The *extension trial* established whether the toddler extended the label to the target object. Here, the experimenter presented the pair of objects from the training phase and requested the target object as she passed them to the toddler (i.e., “Which one can you get? *Fep*!, Can you get it? *Fep*!”). The actor held her hand in between the objects for 10 seconds or until the toddler passed her an object. The *generalization trial* tested whether the toddler could generalize the label to a novel member of the same category. The actor presented the toddler with the pair of objects that differed only in colour from those used in the label training phase and repeated the same request sequence to the toddler. Following the generalization trial, all three phases were repeated for the second set of novel objects and word label.

Following completion of the second block, parents were given the MacArthur-Bates Communicative Development Inventory: Words and Sentences [31] to provide a measure of toddlers’ productive vocabulary. Sixty-one percent (*n* = 44) of the questionnaires were returned.

### Results

**Fig 1:**
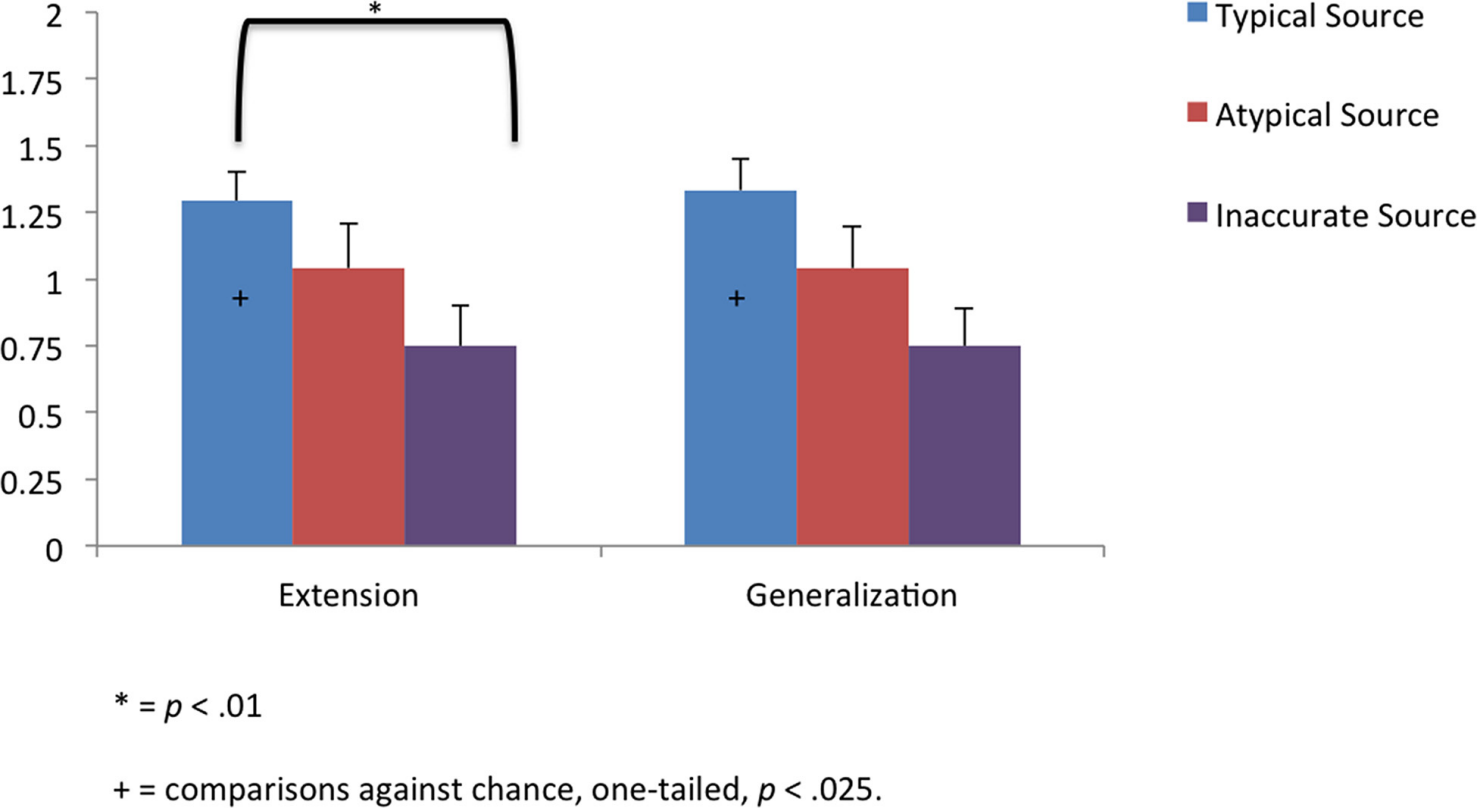
The average number of target object selections (+- SE) during the object label extension and generalization phases (max = 2) made by infants in each group in Experiment 1 (i.e., word labels).

The primary question of interest was whether the nature of a source’s object-directed behavior influenced 24-month-olds’ acquisition and generalization of novel labels. To address this question, we first computed the total number of target choices for the extension and generalization trials for each participant. If the child chose the target object, they were given a score of 1 on a given trial. If the child chose the distractor object or if the child did not choose either object, they were given a score of 0 on a trial. Thus, children received a score of either zero correct, one correct, or two correct for both the extension and generalization trials. We then conducted planned one-way ANOVAs comparing toddlers’ target object choices on each type of trial as a function of group and trial (Fig 1). Note that that this analytic strategy, as opposed to an omnibus analysis, is recommended when specific a priori hypotheses can be formulated [32,33].

Toddlers’ performance on the extension, *F*(2, 69) = 3.53, *η*_*p*_^2^ = .09, *p* = .035, and generalization trials, *F*(2, 69) = 4.57, *η*_*p*_^2^ = .12, *p* = .014, differed as a function of group. Follow-up analyses correcting for multiple comparisons indicated that toddlers in the Typical Source and Atypical Source groups did not differ in their extension or generalization of the novel words (*ps >* .14). Toddlers in the Inaccurate Source group were less likely to extend and generalize the novel words than toddlers in the Typical Source group (*p*s < .01), but not toddlers in the Atypical Source group (*p*s > .13).

Next, we compared toddlers’ performance to what would be expected if they were responding above chance levels for the extension and generalization trials (chance = 1) using directional tests with alpha set at .025. Given that our hypotheses focus on whether toddlers learnt the novel words, we focused on whether toddler’s performance was above chance-levels. Furthermore, given that “no responses” were coded as 0s in our dataset, comparisons to below chance-levels are not meaningful. In the Typical Source group, toddlers’ extension and generalization of the labels to the target objects were both significantly above chance (*ps* < .02). Conversely, in the Atypical Source group and the Inaccurate Source group, toddlers’ performance on both the generalization and extension trials were not significantly greater than chance (*ps* > .083). Thus, even though toddlers’ word learning in the Atypical Source group did not differ from the Typical Source group (as represented in our first set of analyses), the at-chance-level performance suggests that toddlers’ word learning was disrupted, as in the Inaccurate Source group.

### Discussion

The results of the extension and generalization trials of this experiment confirm that 24-month-olds learn new object labels from sources with a history of labeling familiar objects correctly, even when the new labels have been provided in an unconventional labeling frame. These findings extend the results of Namy and Waxman [34] by demonstrating that 24-month-olds will learn a new label that might not be shared by the broader community (because it was offered in an unusual frame) if they are provided with evidence suggesting that doing so would be relevant to the present context. In their study, Namy and Waxman [32] established relevance by providing toddlers with additional experience via training with a source who labeled categories of familiar objects using unconventional frames. In our study, we established relevance of the source simply by demonstrating to toddlers that the source had a history of providing typical labels and acting in conventionally appropriate ways.

The toddlers in the Typical Source group of Experiment 1 showed robust learning. However, their performance appears to be somewhat poorer than the performance of toddlers in previous work using a similar paradigm [29,30,35]. This is likely due to the fact that we used a somewhat unusual labeling frame (i.e., “Look at what I have. *Fep*.”) instead of a typical syntactically informative labeling frame that 24-month-olds have likely come to expect when being taught new words (i.e., “This one is called a *blicket*. Look at the *blicket*”). As noted above, we chose this frame to enable comparisons across all three experiments in this manuscript. Further, the toddlers in our experiment were provided with the novel word-referent pairing three times during training, whereas same-aged toddlers and younger infants in previous research were provided with the novel word-referent pairings five [29] or nine [35] times. Although providing the labels in atypical labeling frames and fewer times during the training period may have resulted in a reduction in toddlers’ performance relative to previous studies, toddlers in the Typical Source group nonetheless showed reliable learning on both extension and generalization test trials.

More importantly, our findings confirm that 24-month-olds are selective word learners. Toddlers are less likely to learn new object labels from a source who either has a history of providing inaccurate labels, or acts on familiar objects in atypical ways. Our finding that toddlers avoid learning words from a source who has previously been shown to provide inaccurate labels is consistent with the body of evidence demonstrating that preschoolers and toddlers avoid learning new word labels that might not be shared by the broader community (for reviews see [1–4]). However, this finding is inconsistent with first-label learning findings of Krogh-Jespersen and Echols [22]. It is possible that the unusual framing used in our study provided toddlers with enough converging evidence to suggest that the label provided by an inaccurate source was unlikely to be relevant and thus, they should not allocate any of their resources towards learning the label. Thus, these findings extend past work by demonstrating that information beyond a source’s reliability or ignorance guides toddlers’ selective learning.

The results of the Atypical Source group remain somewhat unclear. On one hand, the performance of toddlers in the Atypical Source group on the extension and generalization trials did not differ reliably from chance, while on the other hand, toddlers in this group patterned in between the toddlers in the Inaccurate and Typical Source groups. We posit that the source who acted on objects in atypical ways casted some doubt on the potential relevance of the new object label to the broader linguistic community and as a result, toddlers did not form a robust semantic representation of the new labels in this condition.

## Experiment 2

The results of Experiment 1 confirm that toddlers, like preschoolers, are selective word learners. However, the results of the Atypical Source group were somewhat ambiguous (i.e., toddlers in this group patterned in between the toddlers in the Typical and Inaccurate groups). As such, it remains unclear as to whether, or not, toddlers’ selective learning is an all-or-none phenomenon. The possibility that toddlers’ selective learning is not an all-or-none phenomenon is consistent with past work suggesting that children might switch from a semantic-based learning mode to an event-based learning mode in which some memories of the labeling event are retained when they encounter word-referent links that are unlikely to be shared by the broader linguistic community (see also [5]). Sabbagh and Shafman argue that doing so might establish some sort of in-context relevance in the form of a “local” understanding between the child and the labeling source. To test the extent to which toddlers have different modes of learning for information that is not likely to be shared by the broader community, we introduced 24-month-olds to a context in which they would typically show selective learning, but provide them with some reason to suspect a globally irrelevant piece of information might have local relevance. That is, we test whether toddlers, like preschoolers, show some memory for information that they would typically resist learning, if they are given reason to do so.

We addressed this question by drawing upon research demonstrating that, at around 20 months of age, infants begin to restrict the types of symbols they will accept as object names and no longer accept sound labels as relevant, conventional forms [29,30]. Here, we ask whether toddlers will adjust their selective learning and show some memory for sound-object mappings if they are given some reason to suspect that the mapping might have in-context relevance. As in Experiment 1, 24-month-olds were taught a new object label by one of three sources: a source who had previously labeled familiar objects correctly and used them in the appropriate ways, a source who had labeled familiar objects inaccurately, or a source who had labeled familiar objects correctly, but used them in atypical ways. Rather than using words, however, the source labeled novel objects using a sound (e.g., a *ringtone*).

Our predictions were as follows: We did not expect toddlers to learn the sound-object mapping from the inaccurate source since toddlers are not provided with any information at all to suggest that labels from this speaker would be relevant. If anything, the combined behaviours of mislabeling a known object and providing an atypical label should further reinforce the irrelevance of information provided by this source.

Whether toddlers would learn sound-referent mappings from a source who labeled objects correctly and used them in the appropriate ways remained an open question. On the one hand, we expected that toddlers would not learn sound-object mappings from the conventional source, which would be consistent with the evidence that infants’ prior symbolic openness has narrowed by 24-months of age [29,30]. However, we were also open to the possibility that direct evidence of a source’s labeling accuracy might encourage toddlers to accept sound-referent mappings from that source.

Of key interest was whether providing toddlers with information to suggest that atypical object labels would be relevant in the current context would give toddlers reason to acquire such mappings. We reasoned that information about a source’s tendency to use objects in odd ways might signal to toddlers that the person might also label objects in odd ways and, as a result, toddlers might be more inclined to learn sound-object mappings from an atypical source. This line of reasoning is similar to that of Namy and Waxman [34] who demonstrated that infants learned new labels for object categories when the labels were provided in atypical labeling frames (and were thus not clearly object labels) only when they had been exposed to the source labeling familiar object categories using an atypical frame. However, it was also possible that toddlers’ tendency to avoid learning sound-object mappings might be so robust that information about a source’s tendency to act in odd ways would not guide toddlers to adjust their learning of these potentially irrelevant labels.

If toddlers were to learn sound-referent mappings from the atypical source, we were interested in whether such mappings would be consistent with the formation of a semantic-based representation of the mapping (i.e., selecting the target on the extension and generalization trials), or an event-based memory of the labeling episode (i.e., selecting the target on the extension trials only). Due to the potential irrelevance of the sound-object mapping in the broader linguistic community, we expected that toddlers would not form a semantic-based representation of the sound-referent mapping and thus, would not generalize the mapping to another object of like kind. However, if the source’s tendency to act in atypical ways set up an expectation in toddlers that the speaker might label objects in atypical ways as well, it is possible that toddlers might retain some memory of the sound-object mapping for use in the current context. If this were the case, we expected that toddlers would form an event-based memory for the association and thus, would only demonstrate learning on the extension trials.

### Method

#### Participants

Seventy-two 24-month-olds were included in the final sample. Participants were from homes in which English was the primary spoken language, were from varied socioeconomic backgrounds, and were primarily Caucasian (although the latter two factors were not formally assessed). Equal numbers of males and females were randomly assigned to one of three groups: **Typical Source** (*n* = 24; *M* = 24.15 months; *SD* = .85), **Atypical Source** (*n* = 24; *M* = 24.07 months; *SD* = .94) and **Inaccurate Source** (*n* = 24; *M* = 23.98 months; *SD* = 1.00). Toddlers did not differ in age or productive vocabulary size (as assessed by the MCDI: Words and Sentences, [31]) across the three groups (all *p-*values > .82). Eight additional toddlers were tested but were excluded from the final sample due to fussiness (*n* = 7) and experimenter error (*n* = 1).

#### Materials and Procedure

The materials and procedure for this experiment were identical to those used in each group in Experiment 1 with one exception: During the label training and testing phases, sound labels, rather than word labels, were used. That is, when presenting the target object, the experimenter labeled the object with a novel sound (“Look at what I have! [ring]. That’s what we call this one!”). On the extension and generalization trials, the experimenter used a sound label to request the target object (i.e., “Which one can you get? [*ring*], Can you get it? [*ring]*”).

Following completion of the second block, parents were given the MacArthur-Bates Communicative Development Inventory: Words and Sentences [31] to provide a measure of toddlers’ productive vocabulary. Sixty-six percent (*n* = 48) of the questionnaires were returned.

### Results

**Fig 2:**
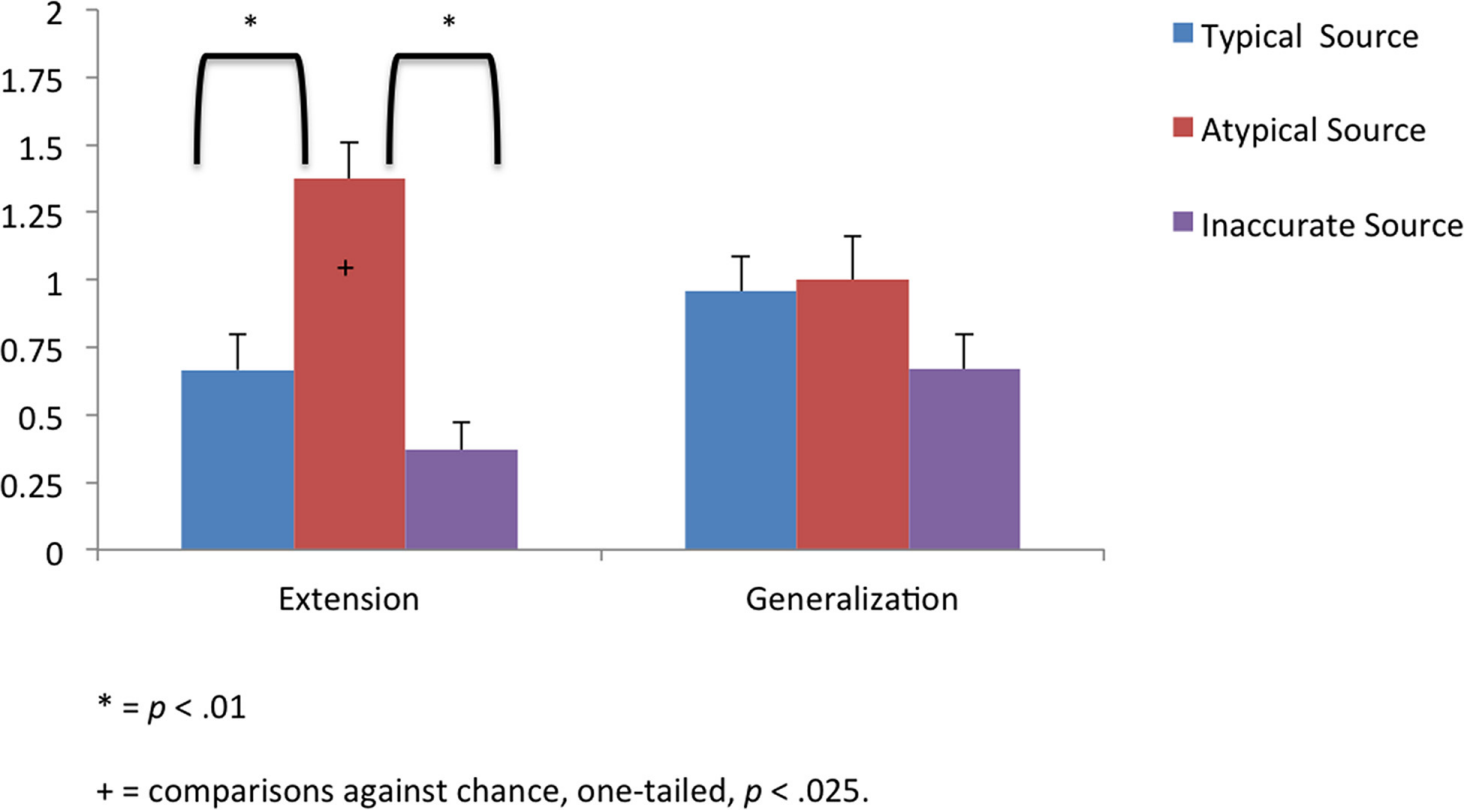
The average number of target object selections (+- SE) during the object label extension and generalization phases (max = 2) made by infants in each group in Experiment 2 (i.e., sound labels).

We first computed the total number of target choices for the extension and generalization trials for each infant (max = 2 for each trial type). As in the first experiment, toddlers were given a 0 if they chose the distractor object, or did not choose either object. As in Experiment 1, we then conducted planned one way ANOVAs comparing toddlers’ target object choices as a function of group for each type of test trial (Fig 2).

Toddlers’ performance on the extension trials differed as a function of group, *F* (2, 69) = 17.82, *η*_*p*_^2^ = .34, *p* < .0001. Follow-up analyses indicated that toddlers in the Atypical Source group were more likely to extend the novel sound label than were toddlers in the Typical Source and Inaccurate Source groups (*ps* < .0001). Toddlers in the Typical Source group and Inaccurate Source group did not differ in their extension of the novel sounds (*ps >* .10). Toddlers in all three groups did not differ significantly in their generalization of the novel sound label to a new exemplar, *F* (2, 69) = 0.19, *η*_*p*_^2^ = 1.69, *p* = .192.

As in Experiment 1, we next compared toddlers’ performance to what would be expected if they were responding above chance levels for the extension and generalization trials (chance = 1, alpha = .025). In the Typical Source and Inaccurate Source groups, toddlers’ extension and generalization of the sound labels to the target objects were not greater than chance *(ps* > .74*)*. Conversely, in the Atypical Source group, toddlers’ extension of the sound label was significantly above chance (*p* <. 01) with generalization at chance-levels, (*p =* 1.0).

### Discussion

The results of this experiment demonstrate that toddlers will show some learning of a sound-object mapping, but only when such mappings are provided by a source who has been shown to act on objects in atypical ways. In contrast, if the source had previously labeled and acted on objects in appropriate ways or had provided inaccurate labels, toddlers showed no evidence of learning sound-object mappings, consistent with previous work revealing that, by 24 months, toddlers restrict the kinds of symbols that they will map onto objects to spoken words only [30,36]. Together, these findings suggest that toddlers will learn sound-object mappings, something that they typically would not learn, if they were provided with information to suggest that the mapping might be relevant within the present context. In our study, toddlers might have expected that a source who acted on objects in odd ways would also refer to objects in odd ways as well.

Our finding that toddlers in the atypical source group showed some learning of sound-object mappings offers the first evidence of any flexibility in 24-month-olds’ unwillingness to learn sound-object mappings. We posit that the familiarization phase in which the actor acted on objects in atypical ways set-up an expectation in toddlers that the source might do other things, such as talk about objects, in atypical ways as well. That is, information about a source’s object-directed behaviour might have established in-context relevance of the atypical sound-object mappings. As a result, toddlers allocated some attention towards the sound-object mapping; a mapping that otherwise would have been ignored. This finding raises the possibility that toddlers may be open to acquiring object labels that are unlikely be shared by the broader linguistic community if they are given some reason to suspect that such labels may have in-context relevance.

However, the fact that toddlers only demonstrated learning of the sound-label associations on the extension trials and not the generalization trials suggests that the toddlers in this group did not form a semantic representation of the sound-object mapping. Instead, we suggest that toddlers’ reliable selection of the target object on the extension trials provides evidence that toddlers only formed an event-based memory of the mapping event in which information about the source is linked with their atypical behaviour (i.e., this person does weird things). One reason why toddlers might form an episode-based memory of the sound-object mapping could be to establish some sort of a local understanding with the source about how objects are referred to in this particular situation. Such a local understanding would be helpful in supporting the ongoing interaction between the child and the speaker (see also [5]).

Our finding that toddlers in the atypical source group showed some memory for the sound-object mappings only on the extension trials suggests that the toddlers in this group formed an object-specific memory of the ‘labeling’ event. One remaining question is whether such mappings are speaker-specific as well. If toddlers encoded aspects of the sound-object mapping event in an effort to establish a local understanding with the source, then the mappings should also be specific to the source who had provided the mapping in the first place. However, it is possible that the toddlers in the atypical source group might have formed an expectation that the learning context was a strange situation in which people do strange things and that they should expect to learn strange names of things, as opposed to a source-specific judgment of contextual relevance. This possibility was explored in Experiment 3.

## Experiment 3

Toddlers were familiarized to a source that acted on objects in an atypical manner. However, unlike Experiment 2, a second source who was not present during the familiarization phase taught toddlers the sound-object mapping (see also [21]). If toddlers’ learning of the sound-object mapping in the second experiment was simply because they believed that doing so would be relevant to the already ‘strange’ context, it was expected that toddlers in this experiment would also demonstrate some memory for the sound-object mapping. If, however, toddlers’ learning of the sound-object mapping in Experiment 2 was due to the formation of a source-specific memory of the ‘labeling’ event, then toddlers in this experiment would not demonstrate any memory for the sound-object mapping in either the extension or generalization trials.

### Method

#### Participants

Twenty-five 24-month-olds were included in the final sample (*M* = 24.41 months; *SD* = 1.33; 15 males). Participants were from homes in which English was the primary language spoken, were from varied socioeconomic backgrounds, and were primarily Caucasian (although the latter two factors were not formally assessed). Toddlers did not differ in age or productive vocabulary size (as assessed by the MCDI: Words and Sentences [31]) from those tested in Experiment 2, *ps* > .67. Four additional toddlers were tested but excluded from the final sample due to fussiness (*n* = 3) and experimenter error (*n* = 1).

#### Materials and Procedure

The materials and procedure for this experiment were identical to those used in the unconventional source group in Experiment 2 with one exception: Following familiarization, the unconventional source left the room and a second source came in to complete the label training and test phases.

### Results and Discussion

As in Experiments 1 and 2, we first computed the total number of target choices for the extension and generalization trials for each infant (max = 2 for each trial type). Comparisons to chance indicated that toddlers’ performance on the extension (*M =* .88; SD = .60) and generalization trials (*M =* 1.04; SD = .67) were not significantly above chance (*ps* > .33). Next, we conducted a planned t-test comparing the number of target choices for toddlers in the atypical source group (Expt 2) to the number of target choices for toddlers in this experiment. Results indicated that toddlers in the atypical source group (Expt 2) extended the novel sound to the target object significantly more often than did toddlers who were familiarized to an atypical source, but were taught a sound label by a second source (Expt 3), *t*(47) = 2.78, *p* < .01, *d* = 0.81. However, toddlers’ generalization of the novel sound labels to the target objects did not differ across the two groups, *t*(46) = 0.19, *p* > .84, *d* = 0.05. Thus, toddlers only showed evidence of forming sound-object mappings when they were taught the mapping by a source who had previously acted on familiar objects in atypical ways.

The finding that toddlers did not learn the sound label in this experiment provides evidence suggesting that the toddlers in Experiment 2 did not learn the sound-object mapping from an atypical source simply because they thought it was a “strange” situation. This finding is consistent with the findings reported by Koenig and Woodward ([21], Experiment 1B) in which a similar manipulation was used to test whether 24-month-olds’ tendency to avoid learning new words from an inaccurate source was a function of the false labeling context, or the inaccurate source. Consistent with our findings, 24-month-olds in Koenig and Woodward’s study were not less likely to learn new object labels if they were introduced to an inaccurate source, but were taught the new labels by a second source. Together with the findings of Experiment 2, these findings suggest that toddlers form object- and speaker-specific memories for sound-object mappings provided by an atypical source. Such mappings are not acquired as a semantic representation of a labeling event, but are indeed likely to be acquired as an event-based memory that will help the child negotiate their current interaction.

## General Discussion

In the present research, we examined the nature of toddlers’ selective learning with a specific focus on the conditions under which 24-month-old children will or will not show selective learning. Specifically, we examined whether: 1) toddlers use information beyond source accuracy and knowledge to guide their selective learning of word labels, and 2) like preschoolers, toddlers’ selective learning is not an all-or-none phenomenon. Three key findings emerged, which we discuss in turn.

First, our findings demonstrate that toddlers are selective learners. As expected, 24-month-olds would not learn new word- or sound-object mappings from a previously inaccurate source. These findings are consistent with the findings of past work demonstrating that preschoolers [7–11] and toddlers [20,21] avoid learning new word-referent links from inaccurate sources. Our finding that toddlers’ learning of new word-object mappings was disrupted in the Atypical Source group (as it patterned in between the Typical Source and Inaccurate Source groups) provides the first evidence that toddlers’ selective word learning extends to contexts beyond source accuracy and knowledge. This finding is consistent with the findings reported by Zmyj and colleagues [23] demonstrating that 14-month-olds do not imitate novel actions provided by an actor who had previously acted on object in atypical ways. However, information about a speaker’s tendency to act on objects in atypical ways did not fully disrupt toddlers’ learning of word labels. It is possible that the fact that the source provided typical labels before acting on object in atypical ways led toddlers not to completely disregard the labels provided by the source. This seems like a reasonable strategy because communication is only successful when individuals use correct word-referent links, however there are often many ways to complete a goal. Toddlers’ tendency to place more weight on a speaker’s labeling accuracy over typicality of actions aligns well with recent evidence suggesting that preschoolers are more likely to imitate novel actions provided by a source who had acted on objects in atypical ways, but was successful at attaining a goal than they were a source who had acted on objects in conventionally appropriate ways, but failed to achieve the goal [17]. An open question is whether toddlers will avoid learning a word label from a source who is unsuccessful at completing action goals, as opposed to someone who simply completes goals in an atypical manner.

Secondly, 24-month-olds did not demonstrate learning of sound-object associations in the Typical Source group. This finding is consistent with the large body of evidence suggesting that toddlers become restrictive in the types of labels they will map onto objects some time during the second year [28–30,36]. Our findings extend this past work by demonstrating that toddlers’ expectations about conventionality of form are robust. Toddlers will not accept sounds as labels even when they are provided with direct evidence that a source labels and acts on objects in typical ways. Although our results do not rule out the possibility that the atypical labeling frame used in this research might have attenuated toddlers’ learning of sound labels from the typical source, the findings from past work lead us to suspect this is an unlikely explanation for our findings. For example, Graham and Kilbreath found that 22-month-olds would not learn a new gesture as a category label even when it was provided in a typical labeling frame (e.g., “This is a [gesture]”).

Thirdly, and most importantly, our findings clearly demonstrate that 24-month-olds’ selective learning is not an all-or-none phenomenon; toddlers will show some learning of a sound-object association if they are given some reason to suspect the association will be relevant within the present context. Past work has demonstrated that preschoolers [5] and toddlers [21] will show some learning of new word-referent links that are unlikely to be shared by the broader linguistic community. Our findings extend this work by providing the first evidence that toddlers will override their symbolic restrictiveness and learn a sound-object association if they are provided with information to suggest such a label might be relevant within the present interaction.

However, toddlers’ learning of the sound-label in the atypical source group was limited in that they did not generalize the sound-label to an object of like kind. This finding is consistent with the possibility that toddlers in this group did not engage in the semantic-based learning mode typical of most word learning contexts and thus, did not form a semantic representation of the association. Instead, we suggest that toddlers formed an event-based memory of the labeling event in which toddlers remembered something about the source’s odd behaviour (e.g., “she does odd things with objects and calls them odd names”). We posit that when the source provided the sound-object association, something toddlers would not typically learn, the fact that the source had previously acted on objects in atypical ways gave toddlers some reason to suspect that the speaker might talk about objects in atypical ways as well. Toddlers’ event-based acquisition of the sound-object association in this context could be useful as it would have provided them with a local convention to communicate (or coordinate their actions) with the atypical source (see also [5,35]).

We speculate that this is an event-based representation rather than a fragile semantic encoding because toddlers did not learn sound-object mappings when they were exposed to an atypical source, but were taught the mapping from a second source (Experiment 3), nor did they show any evidence of learning such mappings from a typical source. One would expect some evidence of extension in both of the above instances if toddlers had formed some form of semantic representation of the sound-object mapping. Thus, we argue that our findings demonstrate that toddlers form object- and speaker-specific memories of sound-object mappings provided by a source who had previously acted on objects in atypical ways. Forming such memories would enable toddlers to establish a sort of local convention with the source (i.e., “I’ll learn this unconventional label for this object while interacting with this person, but only this person”) thereby enhancing their ability to communicate (or coordinate their actions) with someone who appears to intentionally act and label objects in atypical ways.

One question that arises from our findings concerns the extent to which toddlers will maintain sound-object mappings, once acquired, across people and over time. There is clear evidence indicating that infants generalize word-object mappings across people early in development [35,37–40], but see [41] for contexts in which toddlers do not retain word-object mappings over time). However, it is unclear whether unconventional mappings, such as sound-object mappings, are similarly represented. Indeed, other research has found that when children have learned potentially unconventional object labels, the initial mapping reduces significantly after only a minimal delay [4,5,21]. Furthermore, recent research has demonstrated that 18-month-olds treat gestures and words similarly in some word-learning tasks (i.e., mapping, extension) but not in others (i.e., avoidance of lexical overlap) ([39], see also [42] for evidence that 12-month-olds generalize gestures across people). Future work examining whether toddlers maintain the link between an unconventional label and object over time, or whether toddlers would only keep the link for communicating with the speaker in the present context.

Another open question raised by our findings is *why* children are motivated to store some memories of words, or other information that is unlikely to be relevant within the broader community. We, and others, argue that children are motivated to store memories of the labeling event as a means of facilitating the ongoing interaction, perhaps as a sort of local convention (see also [5]). Existing findings support this possibility, as the memories of word-referent links that are unlikely to be shared by the broader linguistic community are object-specific and short-lived [5,21]. However, it remains unclear whether toddlers would maintain the sound-object mappings over a delay if they were provided with reason to suspect that the information would be relevant beyond the present context. Future research could examine whether toddlers would maintain event-based memories of such unconventional object labels if they were told that they would be interacting with the speaker again some time in the future.

Taken together, our findings demonstrate that, like preschoolers, 24-month-olds’ selective word learning is not an all-or-none phenomenon. Toddlers avoid learning new object labels that are unlikely to be shared by the broader linguistic community, but will learn such labels *if* they are provided with reason to believe that the label is likely to be relevant within the current context.
